# Plasmid-normalized quantification of relative mitochondrial DNA copy number

**DOI:** 10.1038/s41598-018-33684-5

**Published:** 2018-10-18

**Authors:** Federica Fazzini, Bernd Schöpf, Michael Blatzer, Stefan Coassin, Andrew A. Hicks, Florian Kronenberg, Liane Fendt

**Affiliations:** 1 Division of Genetic Epidemiology, Department of Medical Genetics, Molecular and Clinical Pharmacology, Medical University of Innsbruck, Innsbruck, Austria; 2 Division of Hygiene and Medical Microbiology, Medical University of Innsbruck, Innsbruck, Austria; 3 Present Address: Unité des Aspergillus, Institut Pasteur, Paris, France; 4 Institute for Biomedicine, Eurac Research, Affiliated Institute of the University of Lübeck, Bolzano, Italy

## Abstract

Alterations of mitochondrial DNA (mtDNA) copy number have been associated with a wide variety of phenotypes and diseases. Unfortunately, the literature provides scarce methodical information about duplex targeting of nuclear and mtDNA that meets the quality criteria for qPCR. Therefore, we established a method for mtDNA copy number quantification using a quantitative PCR assay that allows for simultaneous targeting of a single copy nuclear gene (beta-2-microglobulin) and the t-RNA^Leu^ gene on the mtDNA. We include a plasmid containing both targets in order to normalize against differences in emission intensities of the fluorescent dyes Yakima Yellow and FAM. Applying the plasmid calibrator on an internal control reduced the intra-assay variability from 21% (uncorrected) to 7% (plasmid-corrected). Moreover, we noted that DNA samples isolated with different methods revealed different numbers of mtDNA copies, thus highlighting an important influence of the pre-analytical procedures. In summary, we developed a precise assay for mitochondrial copy number detection relative to nuclear DNA. Our method is applicable to comparative mitochondrial DNA copy number studies since the use of the dual insert plasmid allows correcting for the unequal emission intensities of the different fluorescent labels of the two targets.

## Introduction

Human mitochondrial DNA (mtDNA) is a small (approximately 16,568 base pair), circular and multi-copy genome. It incorporates 37 mitochondrial (mt) genes including 13 coding for essential components of the mitochondrial electron transport chain and of the ATP synthase complex, 22 for mitochondrial transfer RNAs and 2 for ribosomal RNAs^1^. The remaining mitochondrial genes are encoded in the two copies of nuclear DNA (nDNA). The number of mitochondria per cell varies constantly depending on the energy demands, oxidative stress and pathological conditions^2^. Each mitochondrion can contain 2–10 copies of mtDNA and up to 1000 mitochondria are present per cell^3^.

In recent years, the mitochondrial DNA copy number (mtDNA-CN) has been proposed to be a potential biomarker of mitochondrial dysfunction^4^ and studies targeting mitochondrial diversity have increased considerably. This is based on the rationale that the mitochondrial content reflects the energy demand of a cell and is disturbed by an imbalanced energy metabolism^5^. The ratio between mitochondrial and nuclear genomes (mt/nuc) is a suitable measure for the mtDNA content^6^. Several studies have examined the correlation between mtDNA-CN and diverse phenotypes and diseases. An increase of the mtDNA-CN relative to the nDNA is reported in a variety of disease states including acute kidney failure^7^, brain injury^8^, cancer risk^9^ and metabolic disorders^10^. A reduced mtDNA-CN has been observed in Parkinson’s disease^11^, tumour development and progression^12^ as well as aging^13^. Moreover, other studies claimed an increase of mtDNA-CN with age^14^ or a protective effect of higher mtDNA-CN against kidney disease^15^. Both positive and negative associations have been found regarding type 2 diabetes^16,17^ and breast cancer^9,18^.

As recently discussed in the literature, inaccuracies in the methodology for measuring mtDNA-CN might be, in part, responsible for the observed discrepant results^4^. Moreover, the mtDNA-CN measurement can be affected by different pre-analytical factors^19,20^ and reproducibility between different laboratories is challenging especially because the majority of the published studies do not describe their methods in detail.

Quantitative real-time PCR (qPCR) is the current method of choice for mtDNA copy number quantification^4^. The measurement can be carried out also by alternative methods such as next generation sequencing^21^, microarrays^22^ and recently also droplet digital PCR^23^. However, these three approaches are expensive and extremely laborious if applied in epidemiological studies with thousands of samples. Moreover, array-based and sequencing data-based methods are biased by the presence of “wave-like patterns”^24^ that can interfere with an accurate copy number variation detection and cannot be fully corrected bioinformatically^25^.

The aim of this study was to establish a reliable method to quantify mtDNA-CN according to MIQE (Minimum Information for publication of Quantitative real-time PCR experiments) guidelines^26^ that can fulfil requirements for epidemiological studies: fast, automatable for high-throughput, cheap, simple to use and easy to analyse. Taking advantage of recent developments^27,28^ we designed a duplex qPCR-based method with two hydrolysis probes, targeting mtDNA and nuclear DNA, that allows a minimization of the well-to-well variability that occurs when comparing multiple singleplex reactions^29^. In addition, we constructed a dual insert plasmid containing segments of mtDNA and nuclear DNA to correct for inter-assay variability. Furthermore, we investigated the impact of DNA isolation methods on the quantification results of mtDNA-CN.

## Methods

### Samples

All DNA samples were extracted from EDTA blood. Study participants provided informed consent. The extraction methods are described below.

EDTA-blood was collected from:

1. Eighteen healthy donors from the Central Institute of Blood Transfusion and Immunology, Innsbruck, Austria. Blood samples were stored at −80 °C before DNA extraction;
2. Four healthy donors. DNA was extracted immediately after blood collection;
3. 303 hemodialysis patients of the Family Heart and Kidney Study (FHKS). FHKS is an ongoing prospective multicentre cohort study that aims to investigate the genetic variability of selected candidate genes influencing atherosclerotic complication.
4. 176 patients of the German Chronic Kidney Disease (GCKD) study. GCKD is an ongoing prospective observational cohort study including patients with CKD of moderate severity^30,31^. DNA was extracted from frozen ETDA-blood samples.

### Ethical approval and informed consent

The examination protocol of the FHKS study was approved by the Ethics Committee of the Medical University of Innsbruck. The GCKD study was approved by the Ethics Committees of all participating institutions (Friedrich-Alexander-University Erlangen-Nuremberg, Medical Faculty of the Rheinisch-Westfälische Technische Hochschule Aachen, Charité—University Medicine Berlin, Medical Center—University of Freiburg, Medizinische Hochschule Hannover, Medical Faculty of the University of Heidelberg, Friedrich-Schiller-University Jena, Medical Faculty of the Ludwig-Maximilians-University Munich, Medical Faculty of the University of Würzburg). All methods were carried out in accordance with the approved guidelines and the Declaration of Helsinki. Written informed consent was obtained from each study participant.

### DNA extraction methods

The impact of DNA extraction methods on the measurement of the mtDNA-CN was evaluated using four different methods.

- Magnetic beads-based extraction. The EZ1 DNA Blood 200 µl kit (Qiagen Hilden, Germany) and the EZ1 DNA Tissue kit were selected as representatives of the automated kits using silica-coated magnetic beads for extraction. DNA extraction was performed using BioRobot EZ1 Advanced (Qiagen, Hilden, Germany) in sample set (1), (2) and (3) described above. The EZ1 DNA Tissue kit was used to perform a comparison between different lysis buffers. We added an initial erythrocyte lysis step to the original protocol using RBC Lysis Solution (Qiagen, Hilden, Germany). After centrifugation (2 min at 2000  ×  g) the white blood cell pellet obtained was resuspended using two different buffers: the G2 buffer included in the kit and a Pierce RIPA Buffer Lyse (Thermo Fisher Scientific, Waltham, MA, USA). Both buffers were used pure and supplemented with Proteinase K (at 1 mg/ml, Qiagen, Hilden, Germany). Different incubation times were applied to the DNA samples (15 min, 1 h, 3 h, 12 h, 24 h, 48 h and 72 h) before performing the DNA automated extraction. According to the kit instruction manual, G2 buffer lysis time was applied only for 3 h, 12 h, 24 h, 48 h and 72 h. Chemagic Magnetic Separation Module I (PerkinElmer Chemagen Technologie GmbH, Baesweiler, Germany) was used for the DNA extraction of GCKD samples (sample set 4).
- Manual salting out. The DNA was isolated from blood using two different kits: INVISORB Blood Universal Kit (Stratec Molecular, Berlin, Germany) for sample set (1) and (3) and PureGene (Qiagen, Hilden, Germany) for sample set (2). Genomic DNA was isolated from EDTA blood as recommended by the manufacturer. In brief, both protocols comprised a selective erythrocyte lysis, followed by a lysis of remaining cells with an optimized buffer system. Proteins were removed and DNA was recovered by precipitation with isopropanol. DNA was resuspended in the respective elution buffers provided by the kits.
- Phenol-chloroform-isoamyl alcohol extraction (PCI) was used in sample set (1). DNA extraction was performed as described in the previous paragraph but a phenol-chloroform-isoamyl alcohol purification step was introduced before the DNA precipitation step.
- Chelex 100 Resin (BioRad, Hercules, CA, USA) was used in sample set (2). Chelex is a resin binding cations like Mg^++^ which are essential for the activity of DNase. In brief, following the manufacturers protocol, cells were incubated in Chelex solution without further washing steps. This method was chosen in order to get closer to the biological state of the cells and avoid unexpected losses due to washing or precipitation steps.

Purity (absorbance ratio at 260/280) and concentration of DNA samples were assessed using Tecan NanoQuant infinite M200 (Tecan Group Ltd., Männedorf, Switzerland).

In these comparative experiments, the measured values are expressed as means ± standard deviation.

### Duplex real-time PCR assay

A duplex assay based on quantitative real-time polymerase chain reaction (qPCR) was established for measuring the amount of mtDNA-CN relative to the nuclear DNA. This assay allows for the simultaneous targeting of the mitochondrial tRNA^Leu^ (108 bp) and the nuclear single copy gene beta-2-microglobulin, which is an ideal reference for blood cells^11,32–34^(86 bp). The mtDNA target sequence was chosen based on the absence of known phylogenetic polymorphism within the Caucasian population and with a MAF (minor allele frequency) <1% in the global population^35^. The chosen region might incorporate the pathologic A3243G mutation present in 80% of MELAS patients which, however, has only a frequency of 1:10000 in the general population^36^. The specificity of the primer pairs was confirmed *in silico* by Primer Blast^37^. The potential amplification of nuclear insertions of mitochondrial origin (NUMTs) was excluded by deep sequencing of the amplicons using an Illumina MiSeq platform with a minimum coverage exceeding 80,000X. Inspecting the reads for low level admixture of potential NUMTs -specific variants was performed using the mtDNA-Server^38^. The primer and probe sequences were modified from Bai *et al*.^39^. In brief, a region of mtDNA-tRNA^Leu^ was amplified using the forward primer 5′-CACCCAAGAACAGGGTTTGT and the reverse primer 5′-TGGCCATGGGTATGTTGTTA; a region of beta-2-microglobulin (B2M) was amplified using the forward primer 5′-TGCTGTCTCCATGTTTGATGTATCT and the reverse primer: 5′-TCTCTGCTCCCCACCTCTAAGT. Probe sequences were: FAM-5′-TTACCGGGCTCTGCCATCT -BHQ1 for mt-tRNA^Leu^ and Yakima Yellow-5′-CAGGTTGCTCCACAGGTAGCTCTAG-BHQ1 for beta-2-microglobulin. Primers and probes were synthesized by Microsynth AG, Balgach, Switzerland.

The quantitative PCR was performed on the Quantstudio 6 instrument (Thermo Fisher Scientific, Waltham, MA, USA) using the following conditions: 95 °C for 3 minutes for initial polymerase activation, 40 cycles of 95 °C for 15 sec and 60 °C for 1 min. The robustness of the assay was tested using changing primer concentrations (300–600 nM) and annealing temperatures (60–62 °C). PCR amplification was run in a 10 µl reaction consisting of: 5 µl Brilliant III Ultra-Fast qPCR Master Mix with low ROX (Agilent Technologies, Santa Clara, CA, USA), 1 µl mtDNA primers (300 nM each), 1 µl nDNA primers (600 nM each), 1 µl mtDNA probe (300 nM), 1 µl nDNA probe (300 nM) and 1 µl DNA (3–5 ng). Each sample was assayed in triplicate. We used different primer concentrations for the two targets due to amplification advantages of the more abundantly present one. By limiting the amount of mtDNA primers the reaction plateaus earlier and leaves sufficient dNTPs for the amplification of the less abundant target.

### Droplet digital PCR (ddPCR)

To validate our method also with another quantification approach, we measured 52 samples from sample set nr. 1 (see “Methods” section) with ddPCR. We obtained the best results, in term of coefficient of variation and reproducibility, with 600 nM of mt primers, 300 nM of nuclear primers, 250 nM of each probes and 1 ng of DNA per reaction. We performed the experiment on the automated droplet generator and reader from Bio-rad (QX200 system) in 20 μL reactions.

### Plasmid system

**Figure 1:**
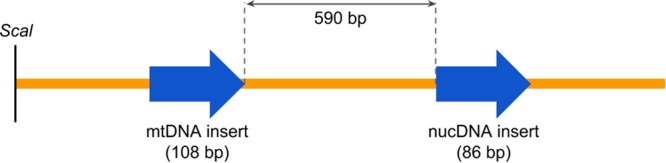
Scheme of the linearized dual insert plasmid (pGEM-T vector, 3194 bp) showing the inserts of human mtDNA and nuclear DNA.

In order to verify whether the two fluorescent dyes present different intensities of fluorescence emission and to normalize for differences between runs (master mix performance, instrument calibration, environmental variability, etc.) a plasmid construct was designed. A portion of mt-tRNA^Leu^ and B2M genes were amplified using the previously reported primers. Two sequential TA cloning reactions were performed to insert the two targets into the pGEM-T vector (Promega Co., Madison, WI, USA). The successful cloning of the two target sequences in a 1:1 ratio was verified by sequencing and PCR. The distance between both inserts was chosen to be greater than 500 bp to avoid the amplification of both targets as one amplicon in the qPCR reaction (Fig. 1).

Plasmids were extracted from NEB Turbo Competent *E. coli* (New England Biolabs, Ipswich, MA, USA) with PureYield Plasmid Midiprep kit (Promega Co., Madison, WI, USA) and the concentration was measured using Qubit fluorometer (Thermo Fisher Scientific, Waltham, MA, USA). The plasmid was linearized by *ScaI* digestion (New England Biolabs, Ipswich, MA, USA) and the linearized plasmid was used as calibrator in all the qPCR experiments.

### Amplification efficiencies

A range of experiments was carried out to test the efficiencies of the PCR reactions. Several serial dilutions of a DNA template and of the plasmid, linear as well as circular, diluted in water or in low-TE (10 mM Tris-HCl and 0.1 mM EDTA) + LPA (Linear Polyacrylamide, 20 µg/ml) were generated to obtain standard curves and determine PCR efficiency (E). PCR efficiency was calculated from the slope of the standard curves using the equation formula: E = 10^−1/slope^ − 1^26^. In order to improve the precision, all reactions for the standard curves were performed in quadruplicates.

### mtDNA copy number calculation

Relative quantification was applied^40^ to calculate the copy number of mtDNA per diploid nuclear (2n) cell:

1 $$2\times {{\rm{E}}}^{-{\rm{\Delta }}{\rm{\Delta }}\mathrm{Cq}}$$

In Equation (1): “Cq” is the quantification cycle, ΔΔCq is (Cq_mt_ −Cq_nuc_)_sample_ − (Cq_mt_ − Cq_nuc_)_plasmid_, “E” is the averaged mean efficiency of the PCR reactions of the two targets^41^ and “2” is to account for the two copies of nDNA in a cell. The mtDNA copy number values were calculated from all three replicates and the mean value was used for downstream analysis. The standard deviation of the ΔCq values among the three replicates was assessed for both targets. Around 5% of the samples had a coefficient of variation (CV) >2% within the triplet reactions and therefore they were excluded from the analysis.

### Inverted probes experiment

To confirm that neither probe sequence nor primer affinity differences could have affected the assay, we performed a validation experiment using the same protocol and primer pairs but inverted labelled probes: B2M FAM-5′-CAGGTTGCTCCACAGGTAGCTCTAG-BHQ1 and mt-tRNA^Leu^ Yakima Yellow -5′-TTACCGGGCTCTGCCATCT-BHQ1. Efficiencies and ΔCq were assessed for the plasmid and for a DNA template using standard curves as described above.

A comparison of 88 samples measured with both probe combinations was carried out.

### Probe batch effect

A comparison experiment between two different probes and primers batches (same sequences and dyes) was carried out. Efficiencies and ΔCq values were assessed for the plasmid and for a DNA template using standard curves. A comparison of 88 samples measured with both probe sets was performed in order to evaluate the correction effect of the plasmid.

### mtDNA copy number accuracy

To evaluate the inter-assay variability a positive control was included in eight independent experiments together with the plasmid. The mtDNA copy numbers of the sample were calculated for the eight plates measured on different days using the ΔCq_plasmid_ to correct the values obtained.

### Statistical analysis

Statistical analyses were performed using RStudio with R 3.2.3 (Vienna, Austria, http://www.R-project.org) and Microsoft Excel (Microsoft Corporation, Redmond, WA). Non-normally distributed data were compared using nonparametric tests (Friedman test and Spearman rank correlation coefficient). A P value ≤ 0.05 was considered statistically significant.

## Results

**Figure 2:**
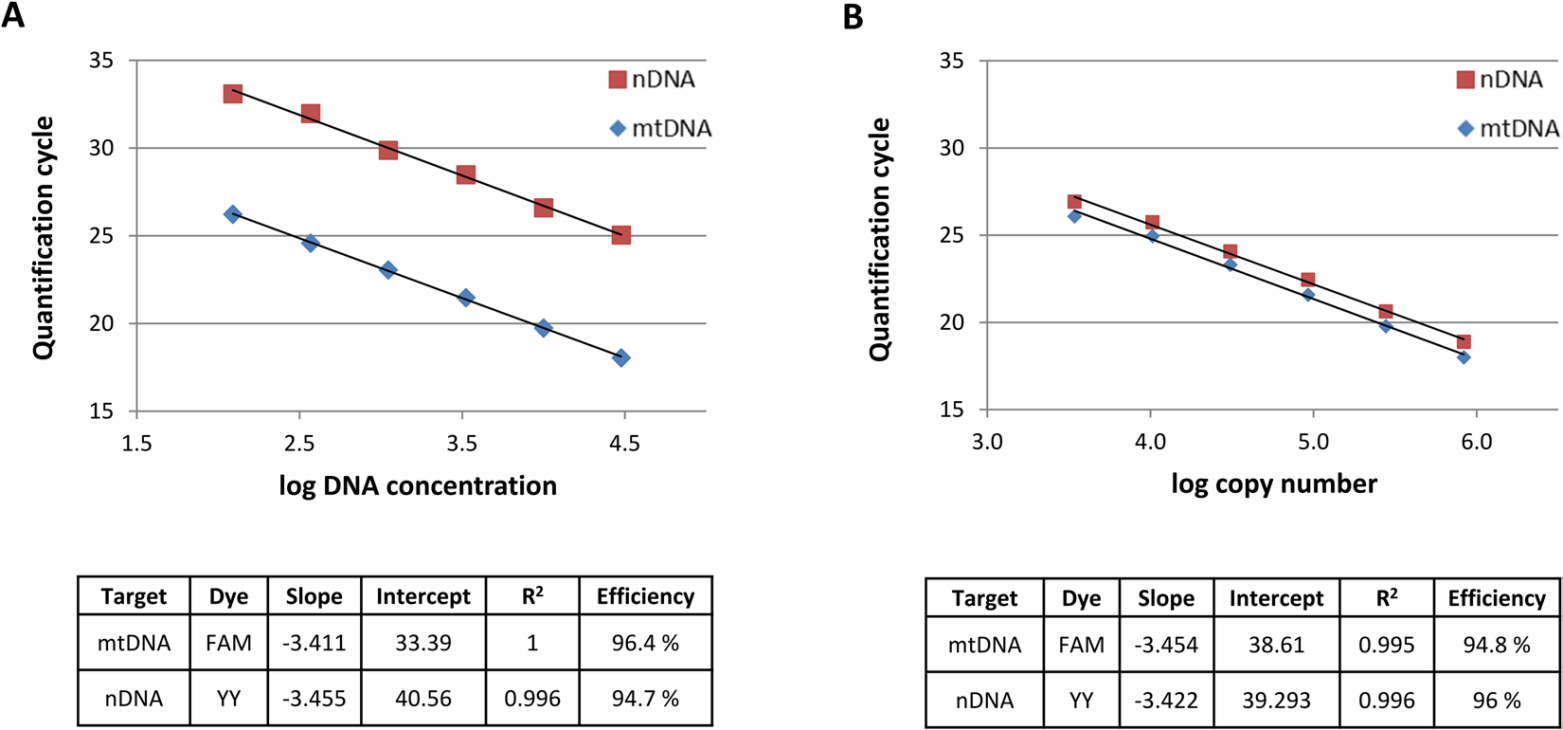
Standard curve regression of a DNA template (**A**) and plasmid DNA (**B**). Quantification cycle (Cq) values on the y-axis and serial 3-fold dilution of the DNA template (**A**) and plasmid DNA (**B**) on the x-axis assayed in quadruplicate by qPCR. Equation of linear regression, R^2^ and efficiencies of both targets are described in the tables. Standard curve of the calibrator plasmid (containing both targets in a 1:1 ratio) revealed differences in emission intensities of the fluorescent dyes Yakima Yellow and FAM.

The efficiency of the assay design was assessed by serial dilutions of DNA standards and of plasmid DNA. The amplification efficiencies obtained from the standard curves were approximately 100% for both targets within the DNA templates (Fig. 2A) as well as within the plasmid (Fig. 2B). All efficiencies comprised values between 94% and 100% with a mean value of 96%.

The standard curves of the calibrator plasmid revealed that the FAM dye emission signal exceeded the Yakima Yellow (YY) signal even though both targets were present in 1:1 ratio on the same plasmid. This resulted in a ΔCq value between FAM and YY probe of up to 1.8 cycles, which would correspond to a 3.5-fold different amount of molecules. Since this difference is caused technically by the differing fluorescence intensities rather than by real difference in target copy number, we subtracted the plasmid ΔCq, i.e. (Cq_mitochondrial_ − Cq_nuclear_)_plasmid_, from each sample.

It has been described by others that using the linearized form of the plasmid is advantageous compared to the circular one^42^. Therefore, we compared the amplification curves of the circular versus the linearized form of the plasmid. No efficiency differences were identified but an average shift in the Cq values of 1.65 ± 0.21 was observed. This is probably due to the supercoiled plasmid form (the dominant template species in the first cycle) and the different accessibility among the two targets. The PCR efficiency of the plasmid suspended in TE + LPA was comparable to suspension in water. Due to these results we used the linearized form of the plasmid for all other experiments.

### Influence of inverted probes on stability of copy number results

**Figure 3:**
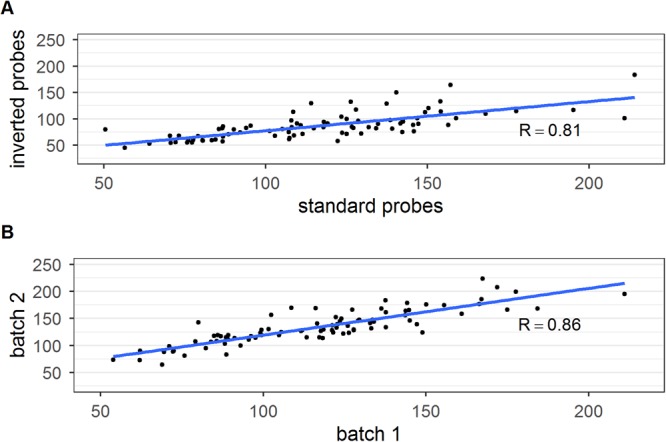
Scatter plots of mtDNA content measurement using standard and inverted labelled probes (**A**) and with two different probe batches (**B**). The correlation is strong according to the Spearman’s rho test (Spearman’s r = 0.81, p < 2.2 × 10^−16^ for inverted vs standard probe, Spearman’s rho = 0.86, p < 2.2 × 10^−16^ for the two different probe batches).

A further evaluation of the assay was performed using the standard PCR protocol with inverted labeled probes (i.e. FAM for nucDNA and YY for mtDNA). The efficiencies of the DNA template and the plasmid DNA were on average 98% and 103%. We found a change in ΔCq (up to 2.2 cycles) between the two probe systems, which, however, did not change the final resulting CN values, because it had been mitigated by our plasmid-based correction. A plate containing 94 samples was analyzed with both probe sets. Six samples were excluded due to high standard deviations in the triplicates. The comparison of mtDNA copy number values in 88 samples between the two probe systems showed consistent results with a strong correlation (Spearman’s r = 0.81, p < 2.2 × 10^−16^). Scatter plot and fitted linear regression curves are shown in Fig. 3A.

### Influence of the plasmid correction factor on the inter-assay variability and effects of probe and primer batches

To validate high throughput screening of large cohorts, we performed a comparison between different probe and primer batches. The amplification efficiencies of a DNA template and the plasmid DNA were both on average 99–100%. When changing probe production batches, we observed a ΔCq change in the sample from 6.51 using the first probe batch to 5.57 using the second batch (ΔCq_batch1_−ΔCq_batch2_ = 0.94). A similar change was detected also in the plasmid, with a change from 1.76 to 0.82 (ΔCq_batch1_−ΔCq_batch2_ = 0.92). This shows that using the plasmid as a correction factor, the “batch effect” can be minimized and the accuracy of the analyses improved. A plate containing 94 samples was analyzed with both probe batches. Six samples were excluded due to high standard deviations in the triplicates. The comparison of mtDNA copy number values in 88 samples between the two probe sets showed consistent results with a good correlation (Spearman’s rho = 0.86, p < 2.2 × 10^−16^). Scatter plot and fitted linear regression curves are shown in Fig. 3B.

No major changes in ΔCq values were detected using different primer batches.

**Figure 4:**
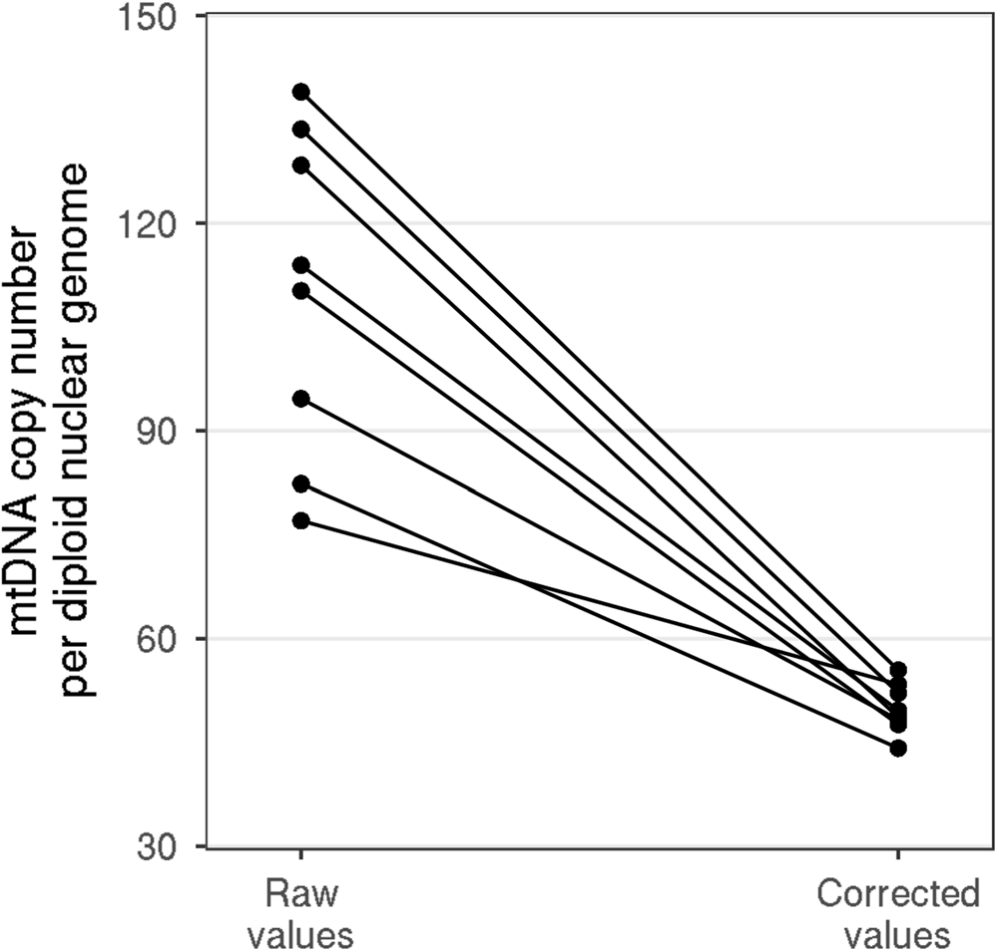
Positive control data for inter-assay reproducibility. One DNA template was included in each of eight independent experiments running on different days. The mtDNA copy number values reached after the calculation using the standard formula are given on the left side. On the right the copy number values obtained using the plasmid correction model. Uncorrected values showed a higher variability (CV was 21% vs 7% of corrected values).

For validation of the inter-assay reproducibility one DNA template was included in each of eight independent experiments running on different days. The calculation of the mtDNA copy number was conducted with and without the use of the plasmid as a correction factor. The CVs were determined for both calculation methods. The use of the plasmid reduced the inter-assay variation from 21% (uncorrected values) to 7% (plasmid corrected value) as shown in Fig. 4.

### Influence of different isolation methods on total extractable mitochondrial genomes

The applicability of the method for the determination of mtDNA copies in a routine epidemiological setting was evaluated using DNA samples that were isolated with different methods. 18 blood samples from 18 healthy volunteer donors were extracted in parallel by three different methods: EZ1 DNA Blood 200 µl kit (EZ1), INVISORB Blood Universal Kit (INV) and phenol-chloroform-isoamyl alcohol extraction (PCI) as described previously.

**Figure 5:**
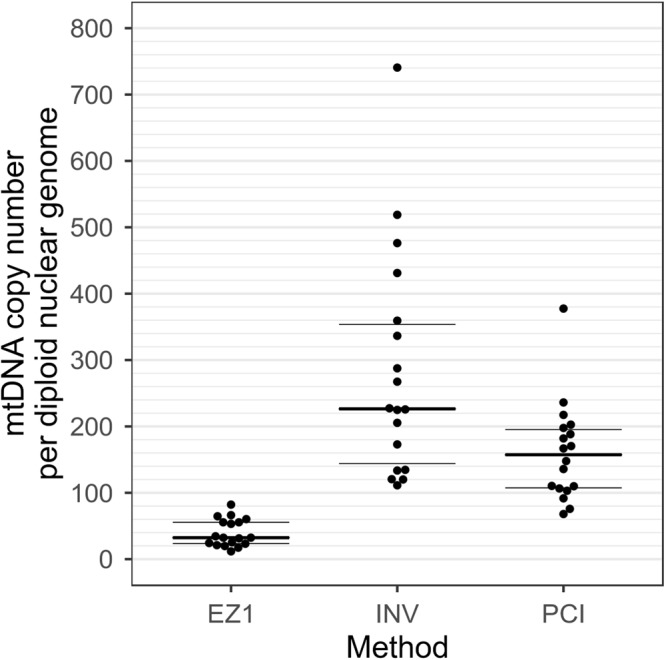
Beeswarm plot illustrating levels of mtDNA extracted by three different DNA extraction methods. MtDNA copy numbers measured in 18 blood samples extracted by EZ1 DNA Blood 200 µl kit (EZ1), a silica-based method, INVISORB Blood Universal Kit (INV), a salting out method, and phenol-chloroform-isoamyl alcohol extraction (PCI). The mtDNA content extracted by INV and PCI is significantly higher than that extracted with EZ1 (Friedman test p-value = 3.419 × 10^−07^). MtDNA content is shown on the y-axis. Lines indicate first, second (median) and third quartile.

We detected significant differences in the mtDNA-CN per 2n genome depending on the extraction methods applied (Friedman test p-value = 3.419 × 10^−07^). The copies of mtDNA/2n averaged 39.5 ± 20 for EZ1, 283 ± 164.6 for INV and 160.4 ± 71.9 for PCI (Fig. 5). Spearman’s rho showed no significant correlation between EZ1 and PCI (Spearman’s rho = 0.14, p-value = 0.56). A moderate correlation was found between INV and PCI (Spearman’s rho = 0.52, p-value = 0.028) and INV and EZ1 (Spearman’s rho = 0.47, p-value = 0.049). A subgroup of samples (n = 52) were typed in parallel with ddPCR. We achieved an average of 16100 ± 1700 accepted droplets per sample. Three samples were excluded because of weak signal. The results showed a strong correlation between qPCR and ddPCR results (Spearman’s rho = 0.80, p = 5.47 × 10^−12^).

**Figure 6:**
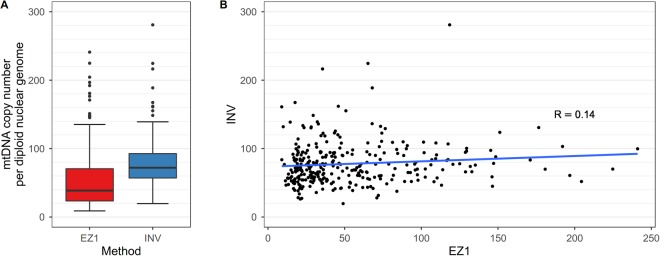
Box plot (**A**) and scatter plot analysis (**B**) illustrating mtDNA content measured in 303 blood sample extracted by two different DNA extraction methods. DNA was extracted by EZ1 DNA Blood 200 µl kit (EZ1) and INVISORB Blood Universal Kit (INV). MtDNA content (as described in the text) is shown on the y-axis. We found only a weak correlation between the two methods (Spearman’s r = 0.14, p = 0.015).

A difference in quantification results depending on the extraction methods was identified also in a subsequent experiment on a larger sample size. We measured the mtDNA-CN in a cohort study of 303 probands of the FHKS study where the DNA was extracted from blood using two methods (EZ1 and INV). The measurements based on INV were higher than measurements based on EZ1, as showed in Fig. 6A. There was only a weak correlation between the two methods (Spearman’s rho = 0.14, p = 0.015). Scatter plot and fitted linear regression curves are shown in Fig. 6B.

### Extractability and reproducibility of total DNA extraction using different extraction methods

To check the intra-assay repeatability of the three different extraction methods we investigated parallel measurements on total DNA from 4 healthy donors. Each blood sample was divided in 45 identical aliquots (300 µl each): 15 of them were extracted by EZ1, 15 with PureGene kit (PG) and 15 with Chelex resin.

**Figure 7:**
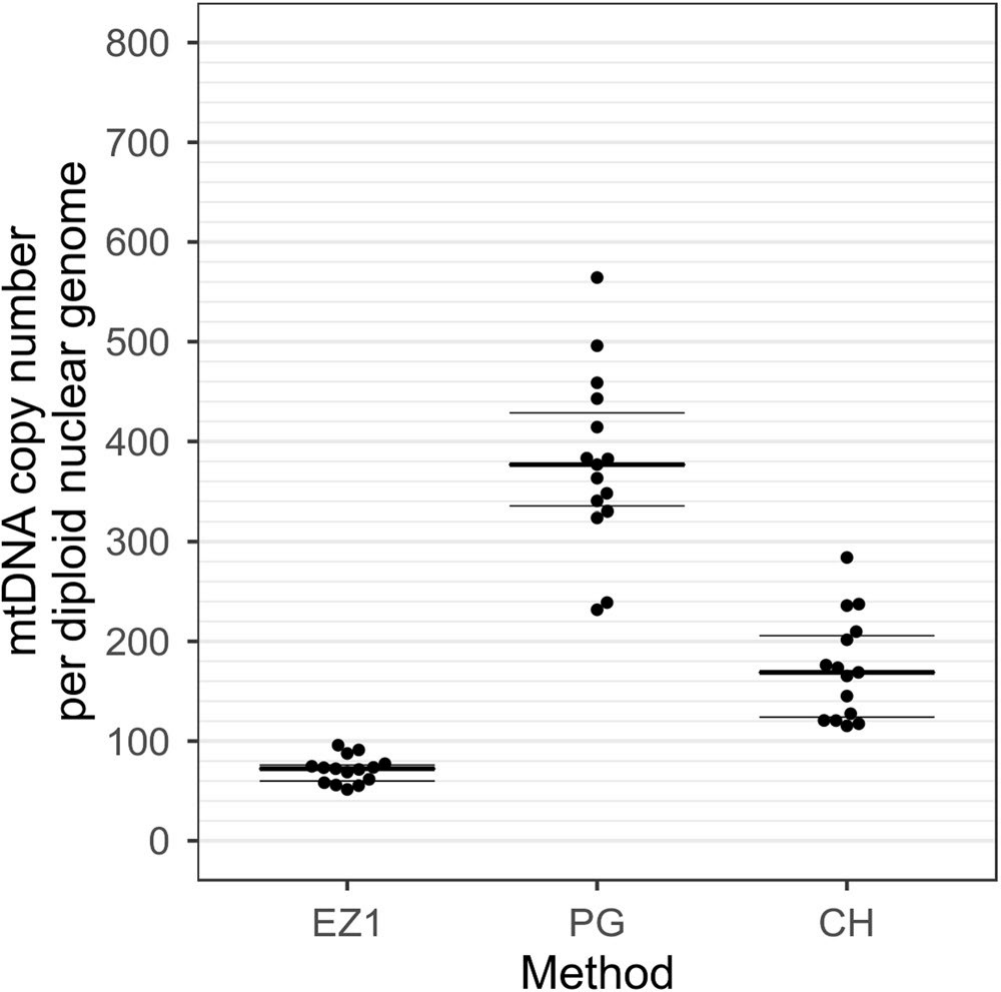
Chart showing mtDNA content measured in 45 identical aliquots obtained from the same blood sample extracted in parallel using three different DNA extraction methods. DNA was extracted using EZ1 DNA Blood 200 µl kit (EZ1), PureGene kit (PG) and Chelex resin (CH). Higher values and considerable variability were detected with the two manual methods (INV 332.1 ± 130.3 and Chelex 206.5 ± 55.2 mtDNA copies, average CV were 27.5% and 24.6% respectively) than in the automated method EZ1 (averaged 63.8 mtDNA copies ±15.7, average CV was 15%). The quantitative mtDNA content is shown on the y-axis.

Automated EZ1 extraction showed the lowest copy number values (averaged 63.8 mtDNA copies/2n ± 15.7), but also the smallest variability within the aliquots (average CV was 15%). Higher values and considerable variability were detected with the other two manual methods (PG 332.1 ± 130.3 and Chelex 206.5 ± 55.2 mtDNA copies/2n, average CV were 27.5% and 24.6% respectively). A representative sample is displayed in Fig. 7.

The PCR efficiencies of eight aliquots carrying the largest variability were checked by standard curves to exclude a bias in the PCR reactions such as the presence of PCR inhibitors in the sample. The PCR efficiencies were calculated from the slope of the standard curves obtained by serial dilution of the samples using the formula previously reported^26^. All but one of the efficiency values were in a range from 95% to 102%.

### Influence of cell lysis buffer on the target accessibility

**Figure 8:**
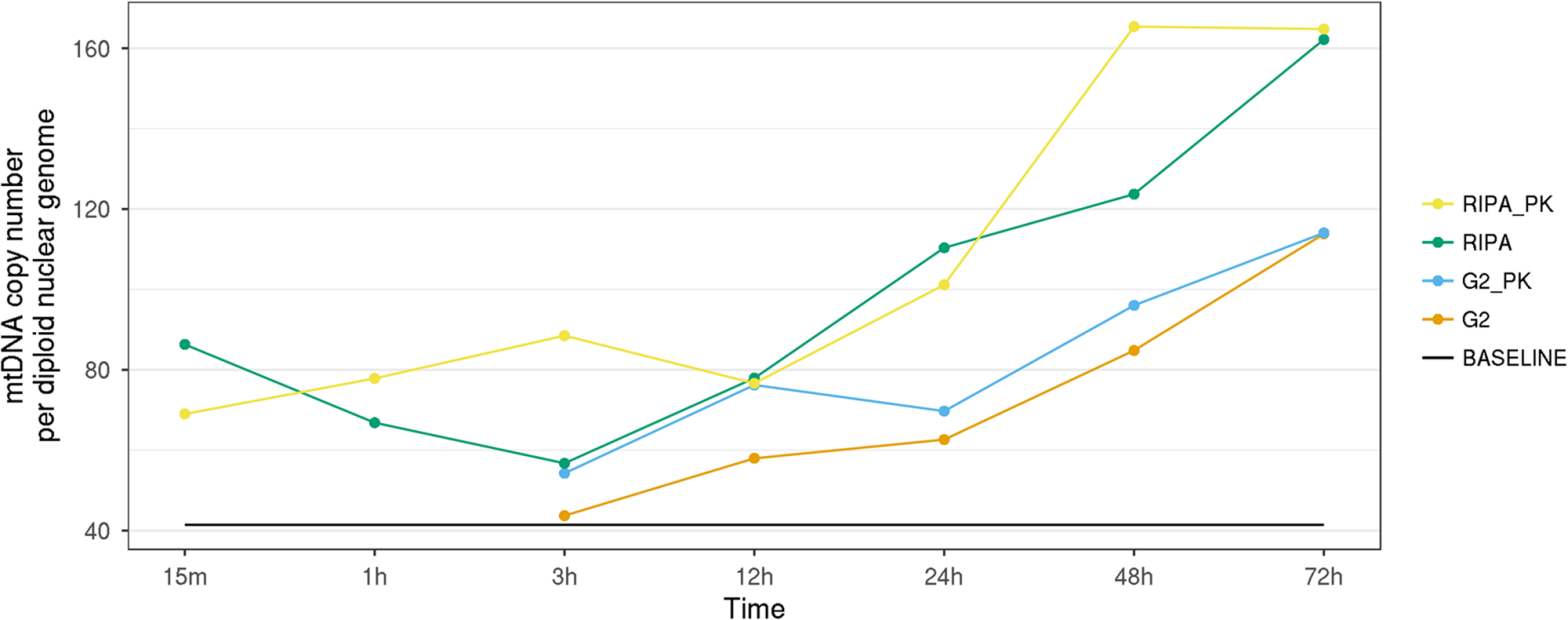
Time curve showing mtDNA content measured in a blood sample extracted by BioRobot EZ1 Advanced after incubation using 4 different lysis buffers. The buffers used were G2 (included in the EZ1 Tissue kit) and Pierce RIPA Buffer Lyse (Thermo Fisher Scientific, Waltham, MA, USA). Both buffers were used pure and in combination with Proteinase K (PK). The baseline is obtained using the standard EZ1 tissue kit protocol without incubation.

In order to verify whether these discrepancies in the mtDNA-CN results were due to a different efficacy of cell membrane lysis techniques during the standard DNA extraction protocol (potentially resulting in unpredictable losses of mtDNA or mitochondrial particles), an experiment with four different lysis buffers was performed and the mtDNA-CN values were measured at six different incubation time points. The extractions were then completed using EZ1 DNA Tissue kit and the BioRobot EZ1 Advanced. The reference baseline for mtDNA-CN retrieved from the EZ1 tissue kit was at 41.4 copies per diploid genome. All used lysis buffers showed an increase of accessible mitochondrial genomes over time. We reached the highest values after 72 hours of incubation with RIPA buffer used in combination with proteinase K (Fig. 8).

A subgroup of samples extracted in parallel with EZ1 Tissue kit after 72 hours of incubation with RIPA + PK and PureGene showed very close CV values (PG 16.16% vs RIPA + PK 17.48%) but no correlation (Spearman rho’s = 0.1, p = 0.95).

## Discussion

The intent of this study was to design a high-throughput assay to allow precise and reliable quantification of mtDNA-CN defined as the ratio of molecules per diploid cell. The growing interest in mtDNA-CN variations and the conflicting results in the recent literature reveal the need to establish a solid and cost-effective method for this measurement. We show that the use of a dual insert plasmid as a calibrator allows the correction against unequal emission intensities of the differently fluorescence labelled probes between different runs and improves the accuracy of the results. This approach combined with a duplex qPCR assay does not require external standards and is easily applicable in large-scale epidemiological studies. Moreover, it allows cost reduction and an increased reproducibility of measurement while keeping the intra- and inter-assay variability low. Furthermore, our results showed a strong correlation with droplet digital PCR. Additionally, we found convincing evidence that mtDNA content measurement is strongly influenced by the DNA isolation method as already suspected by Guo *et al*.^43^. However, this previous study was limited by a low number of samples (n = 8) and only two DNA extraction methods (silica-based column and phenol-chloroform-isoamyl alcohol).

In many publications qPCR approaches have been used to measure mtDNA-CN. First, the literature data are contradictory in terms of the association drawn by the particular study and second, even the real mtDNA copy number stated for a specific cell type is not reproducible among the different investigations. This lack of reproducibility across laboratories^44^ could be due to several issues such as inappropriate primers sets, dilution bias^34^, lengths of amplicon^33^ but also – as we investigated extensively in the current study – different primer and probe batches, instrument calibration problems, different isolation procedures or variations within dye emission intensities. Our approach addressed the MIQE quality criteria. In particular, it was challenging to design specific mtDNA primers since more than 95% of the mitochondrial genome is duplicated into the nuclear genome^4^. To our knowledge, we are the first to confirm the specificity of our mtDNA primers by ultra-deep-sequencing: a perfect alignment of all reads with a coverage of over 80,000X demonstrated the unique amplification of mitochondrial genomes and the complete absence of co-amplified “nuclear insertions of mitochondrial origin” (NUMTs).

The analysis of the plasmid quantification results showed an unexpected ΔCq among the two targets. Despite the same amount of starting copies in each plasmid (1:1 ratio, verified also by sequencing) and a perfect equality in PCR efficiencies, the two amplification curves did not overlap. This resulting ΔCq is very likely due to differences in the probe fluorescence or a bias how the probe signals are detected. An identical, in terms of magnitude, “artificial” increase in ΔCq has been observed also in the DNA samples. For this reason, we modified the standard formula for copy number calculation applying the (Cq_mt_ − Cq_nuc_)_plasmid_ to correct the (Cq_mt_ − Cq_nuc_)_sample_. Based on eight independent experiments we demonstrated that the use of the plasmid considerably reduces the inter-assay CV and therefore improved the accuracy of the analyses. This demonstrates that differences in fluorescence dye emission intensity and/or uncalibrated emission filter settings within the detection instrument are likely to occur for any used combination of dye and platform. Therefore, we strongly recommend the inclusion of a plasmid calibrator for any individually designed multiplexed qPCR assay.

During the validation process we found evidence that DNA isolation methods severely affects the measurement of mtDNA by altering the mtDNA/nDNA ratio. Significant differences in mtDNA-CN values between DNA isolation methods and no or little correlations between the different methods revealed a substantial pre-analytical bias of mtDNA-CN determination. A pronounced variability was found also within aliquots of the same blood sample extracted repeatedly with different DNA extraction methods (INV and Chelex) albeit the PCR efficiencies were constantly around 98% for all the samples. This convincingly demonstrated that the differences in the mtDNA-CN values cannot be explained by differences in qPCR efficiencies but by a strong variability of molecules present in a single aliquot probably caused by the pre-analytical steps.

In our study, the manual salting out-based methods PG and INV always resulted in the highest values but also the largest absolute and relative variability. The variability among aliquots was probably due to a problematic DNA elution step. The presence of undissolved DNA clumps could have altered the mtDNA-CN measurement. In contrast, Nacheva and colleagues^20^ found that phenol-based DNA extraction led to higher number than Puregene (salting-out method). However, they measured the copy number variation using sequencing data in fresh frozen brain samples.

Additionally, by applying Chelex extraction we recovered lower numbers of mitochondrial genomes than with the salting-out approach although this method does not require any washing step and should therefore represent the real mtDNA/nDNA ratio within a cell. Using EZ1 as representative of a magnetic bead-based extraction, we obtained the lowest values together with the smallest absolute and relative variability among aliquots. It is known that longer DNA fragments are better retrieved during the magnetic bead-based DNA extraction^45^, therefore we assume that the beads preferentially bind nuclear DNA as compared to the smaller mtDNA, which is more easily lost during the washing steps. Moreover, automated EZ1 extraction procedure led to higher reproducibility of the results compared to manual methods. This was probably due to the manual handling procedure.

Furthermore, the DNA extraction process of mtDNA may be impeded compared to nuclear DNA due to the fact that the molecules are surrounded by a double bilayer where one of them incorporates extensive invaginations as compared to the bilayer of the nucleus. DNA samples incubated at different time points with four different lysis buffers indicated that a more aggressive buffer combined with a longer incubation allowed the retrieval of a larger numbers of mtDNA molecules. We therefore conclude that mtDNA-CN values measured might not reflect the precise biological ratio. With the currently available extraction protocols, it might well be that neither a complete extraction of total mtDNA nor a precise biologically representative ratio of both types of DNA molecules is feasible. Based on these findings it is essential that automated extraction protocols with low aliquot variability should be used for mtDNA-CN studies. Furthermore, it is crucial that all samples are processed under the same conditions and only one type of DNA extraction method must be used within a study cohort or in studies between different cohorts. This should be applied to prevent artificial associations based on different extraction methods between the two observed study groups rather than due to real associations. Similar observations have been made recently by our group in a study that revealed different association with relative telomere length measurements depending on the DNA extraction method applied^46^. Low numbers of mtDNA copies that might not be representative for the real mtDNA-CN such as we experienced with EZ1 could result in false negative results. Hence, a limitation is a potential loss of power of the study rather than a false positive association. The chance for missing a biological difference between two groups can be overcome by increasing the sample size which might at least in part compensate a larger measurement error.

In conclusion, we developed an accurate assay for mitochondrial copy number detection including a plasmid for correction of the intra-assay and inter-assay variability. Furthermore, we discovered that the different DNA extraction methods selectively isolate different amounts of the two types of DNA molecules in a sample. It is therefore crucial to take into account these differences before performing a comparative mitochondrial copy number study. To have reproducible and reliable results, an automated extraction method is strongly recommended and the same protocol should be followed in the entire study.

## Data Availability

The datasets generated during and/or analysed during the current study are available from the corresponding author on reasonable request.
